# Supplementary material for: The SARS-CoV-2 mRNA-1273 vaccine elicits more RBD-focused neutralization, but with broader antibody binding within the RBD
Source: bioRxiv. 2021 Apr 14:2021.04.14.439844. Preprint. [Version 1] doi: 10.1101/2021.04.14.439844 (PMC8057239; doi:10.1101/2021.04.14.439844)
Supplement: 1 — Fig. S1. Raw ELISA and neutralization curves of mRNA-1273 serum samples before and after depletion of RBD-binding antibodies. Fig. S2. Schematic of the deep mutational scanning approach used to quantify the effects of RBD mutations on antibody escape. Fig. S3. FACS gating strategy to select yeast cells that express RBD mutants with reduced binding by serum antibodies. Fig. S4. Site- and mutation-level correlations between serum-escape measurements for each replicate library. Fig. S5. Binding-escape maps for the day 119 sera from all 14 individuals who received the 250 μg vaccine dose. Fig. S6. Escape maps from individuals who received the 100 μg dose of mRNA-1273 119 days post-vaccination largely resemble those of individuals who received the 250 μg dose. Fig. S7. Complete escape maps six representative convalescent plasmas from the day 100–150 cohort. Fig. S8. Escape maps and effects of individual RBD mutations on neutralization for representative samples from vaccinated and convalescent individuals. Fig. S9. Full neutralization curves for all assays testing how RBD mutations affected viral neutralization. Table S1. Serum neutralization titers pre- and post-depletion of RBD-binding antibodies. Table S2. Information on FACS sorting to select cells expressing RBD mutants with reduced binding by sera from vaccinated individuals. Table S3. Measurements of effects of all amino-acid mutations to the RBD on serum binding. [file NIHPP2021.04.14.439844-supplement-1.pdf]

bioRxiv preprint doi: https://doi.org/10.1101/2021.04.14.439844; this version posted April 14, 2021. The copyright holder for this preprint (which was not certified by peer review) is the author/funder, who has granted bioRxiv a license to display the preprint in perpetuity. It is made available under aCC-BY 4.0 International license.

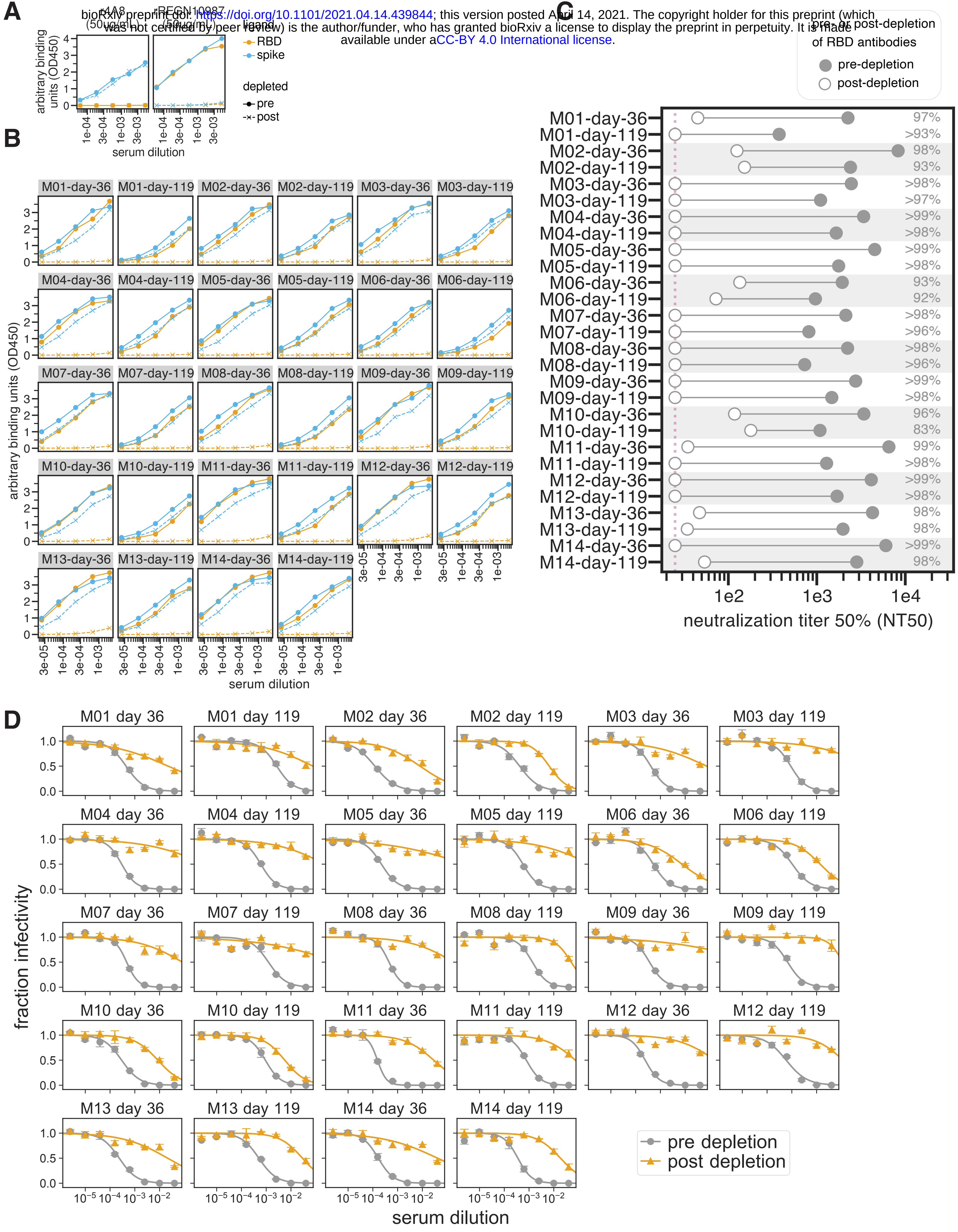

bioRxiv preprint doi: https://doi.org/10.1101/2021.04.14.439844; this version posted April 14, 2021. The copyright holder for this preprint (which was not certified by peer review) is the author/funder, who has granted bioRxiv a license to display the preprint in perpetuity. It is made available under aCC-BY 4.0 International license.

Fig. S1. Raw ELISA and neutralization curves of mRNA-1273 serum samples before and after depletion of RBD-binding antibodies (A) Effect of RBD antibody depletion on binding to RBD and spike by "synthetic sera" comprised of pre-pandemic pooled serum with the NTD-targeting antibody r4A8 (*21*) or RBD-targeting antibody rREGN10987 (*49*). Antibodies were added to pre-pandemic serum at 50 μg/mL. The x-axis indicates the dilution factor of the serum+antibody mix, and the y-axis is the ELISA reading at each dilution. These controls were previously used in (*15*), and demonstrate that the depletions effectively remove RBD-targeting antibodies but not antibodies targeting other epitopes such as the NTD. **(B)** Raw ELISA binding curves of sera to RBD and spike before and after depletion of RBD-binding antibodies. Legend for panels **(A)** and **(B)**: orange is RBD binding, blue is spike binding; filled circles with solid lines represent pre-depletion, and x's with dashed lines represent post-depletion of anti-RBD antibodies. **(C)** Neutralization titer 50% (NT50) of vaccine-elicited sera pre- and post-depletion of RBD-binding antibodies, shown in filled and open circles, respectively. All neutralization assays were performed with SARS-CoV-2 spike D614G-pseudotyped lentiviral particles. Two time points were assessed per individual, at day 36 and day 119 post-dose 1 of vaccination. The limit of detection is shown as a dashed pink vertical line. The percent neutralization due to RBD-binding antibodies are shown at right. **(D)** Raw neutralization curves for sera before (gray) and after (orange) depletion of RBD-binding antibodies. Each assay was performed in technical duplicate, and points show the mean and standard error of the replicates.

bioRxiv preprint doi: https://doi.org/10.1101/2021.04.14.439844; this version posted April 14, 2021. The copyright holder for this preprint (which was not certified by peer review) is the author/funder, who has granted bioRxiv a license to display the preprint in perpetuity. It is made available under aCC-BY 4.0 International license.

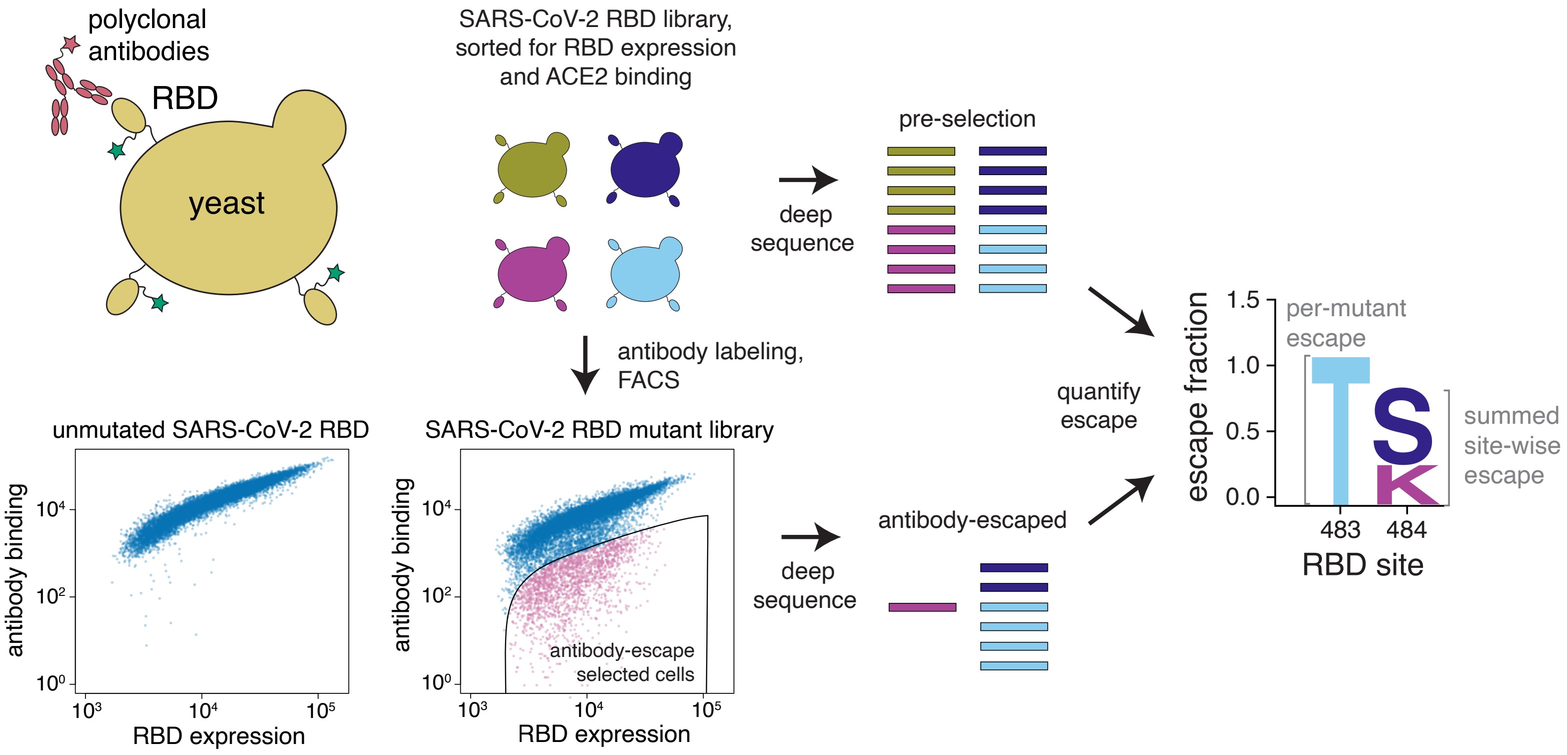

Fig. S2. Schematic of the deep mutational scanning approach used to quantify the effects of RBD mutations on antibody escape. The RBD is expressed on the surface of yeast (top left). Flow cytometry is used to quantify both RBD expression (via a C-terminal MYC tag) and antibody binding to the RBD protein expressed on the surface of each yeast cell (bottom left). A library of yeast expressing RBD mutants was incubated with polyclonal serum and fluorescence-activated cell sorting (FACS) was used to enrich for cells expressing RBD that bound reduced levels of serum antibodies, as detected using an IgA+IgG+IgM secondary antibody. Deep sequencing was used to quantify the frequency of each mutation in the initial and "antibody escape" cell populations. We quantified the effect of each mutation as the "escape fraction," which represents the fraction of cells expressing RBD with that mutation that fell in the "antibody escape" FACS bin. Escape fractions are represented in logo plots, with the height of each letter proportional to the effect of that amino-acid mutation on antibody binding. The site-level escape metric is the sum of the escape fractions of all mutations at a site. Experimental and computational filtering was used to remove RBD mutants that were misfolded or unable to bind the ACE2 receptor (see **Methods**).

bioRxiv preprint doi: https://doi.org/10.1101/2021.04.14.439844; this version posted April 14, 2021. The copyright holder for this preprint (which was not certified by peer review) is the author/funder, who has granted bioRxiv a license to display the preprint in perpetuity. It is made available under aCC-BY 4.0 International license.

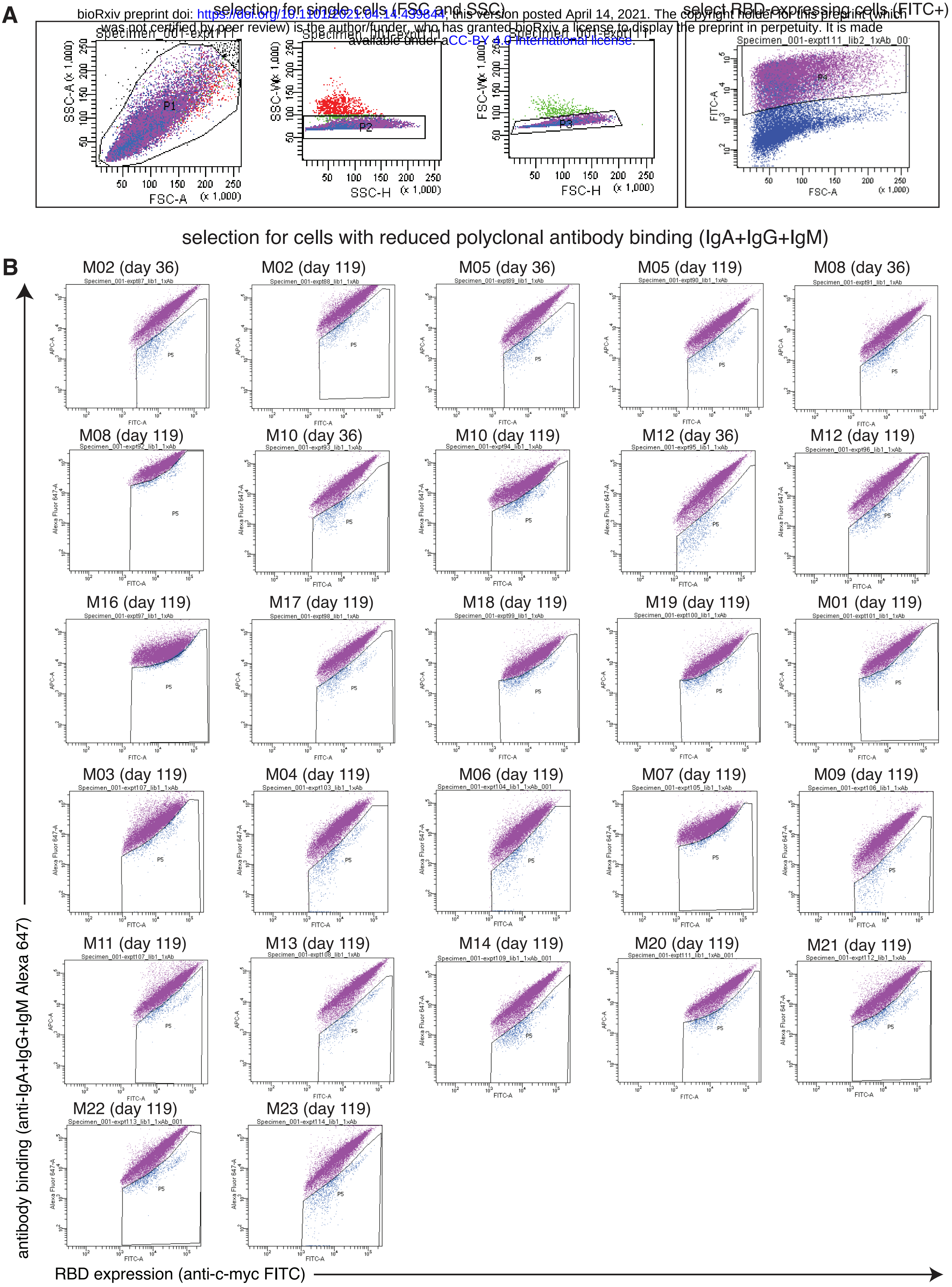

bioRxiv preprint doi: https://doi.org/10.1101/2021.04.14.439844; this version posted April 14, 2021. The copyright holder for this preprint (which was not certified by peer review) is the author/funder, who has granted bioRxiv a license to display the preprint in perpetuity. It is made available under aCC-BY 4.0 International license.

Fig. S3. FACS gating strategy to select yeast cells that express RBD mutants with reduced binding by serum antibodies. (A) Representative plots of nested FACS gating strategy used for all serum selection experiments to select for single cells (SSC-A vs. FSC-A, SSC-W vs. SSC-H, and FSC-W vs. FSC-H) that also express RBD (FITC-A vs. FSC-A). **(B)** FACS gating strategy for one of two independent libraries to select cells expressing RBD mutants with reduced binding by polyclonal sera (cells in blue). Gates were set manually during sorting. Selection gates were set to capture ~5% of the RBD+ library. The same gate was set for both independent libraries stained with each serum, and the FACS scatter plots looked qualitatively similar between the two libraries. For information on the fraction of library cells that fall into each selection gate, see **Supplementary Table 2.**

bioRxiv preprint doi: https://doi.org/10.1101/2021.04.14.439844; this version posted April 14, 2021. The copyright holder for this preprint (which was not certified by peer review) is the author/funder, who has granted bioRxiv a license to display the preprint in perpetuity. It is made available under aCC-BY 4.0 International license.

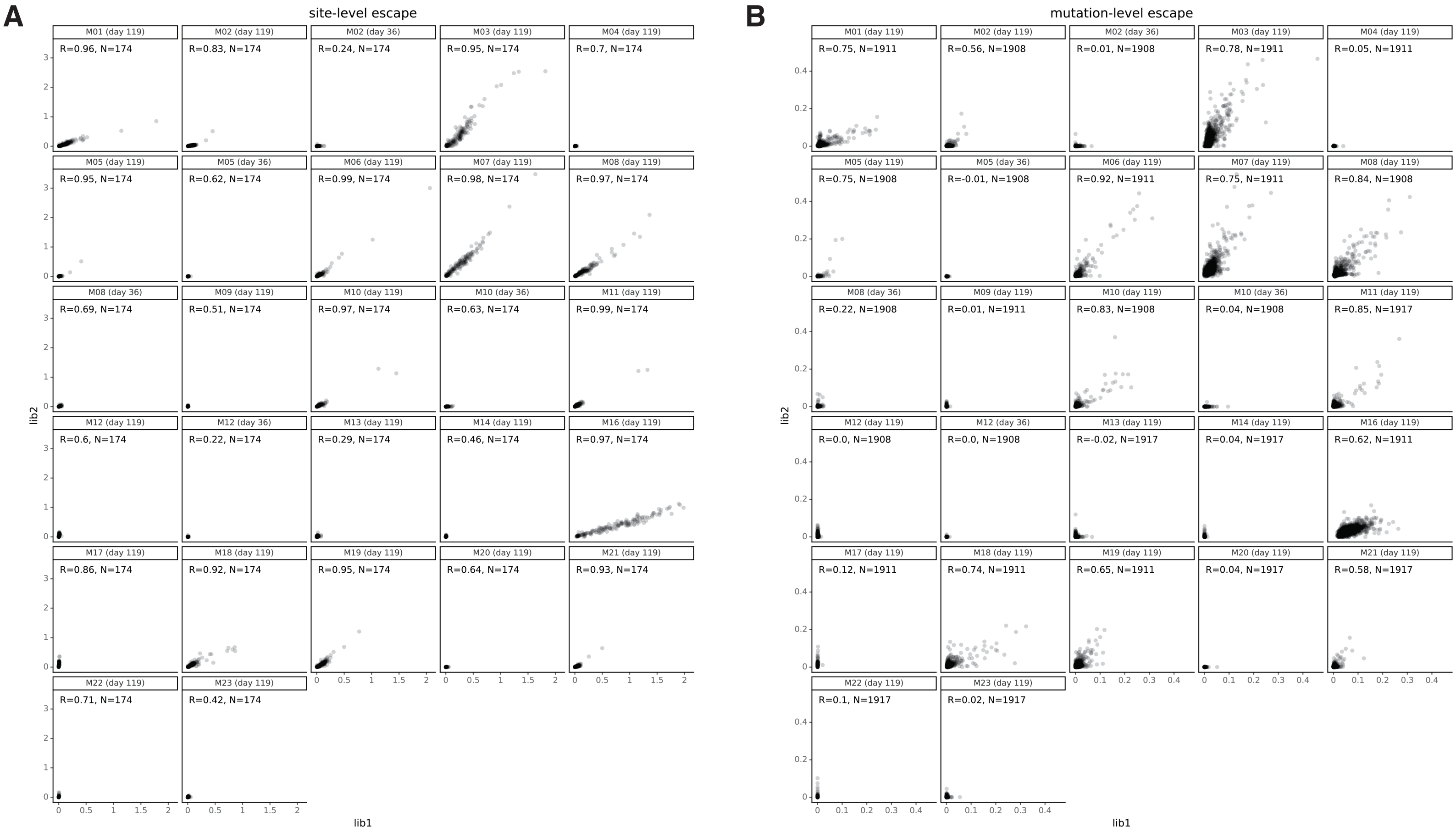

Fig. S4. Site- and mutation-level correlations between serum-escape measurements for each replicate library. Correlation plots of site- or mutation-level escape for each of the two independent RBD mutant libraries for each serum sample. Each point represents one site in the RBD in **(A)** or a different mutation in **(B)**.

bioRxiv preprint doi: https://doi.org/10.1101/2021.04.14.439844; this version posted April 14, 2021. The copyright holder for this preprint (which was not certified by peer review) is the author/funder, who has granted bioRxiv a license to display the preprint in perpetuity. It is made available under aCC-BY 4.0 International license.

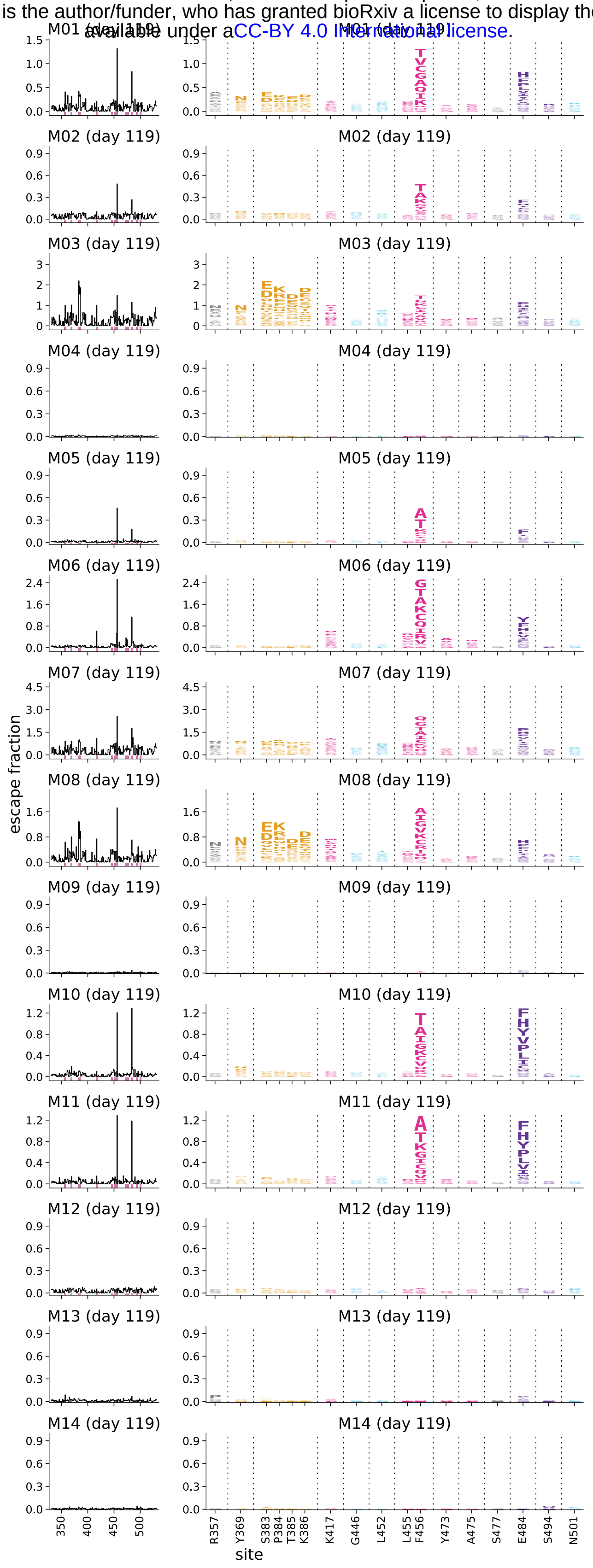

Fig. S5. Binding-escape maps for the day 119 sera from all 14 individuals who received the 250 μg vaccine dose. Complete escape maps for the day 119 sera from the 14 individuals who received the 250 μg dose of mRNA-1273; note that a subset of these sera are also shown in **Fig. 2C**. RBD sites are colored according to epitope, as in **Fig. 2A**.

bioRxiv preprint doi: https://doi.org/10.1101/2021.04.14.439844; this version posted April 14, 2021. The copyright holder for this preprint (which was not certified by peer review) is the author/funder, who has granted bioRxiv a license to display the preprint in perpetuity. It is made available under aCC-BY 4.0 International license.

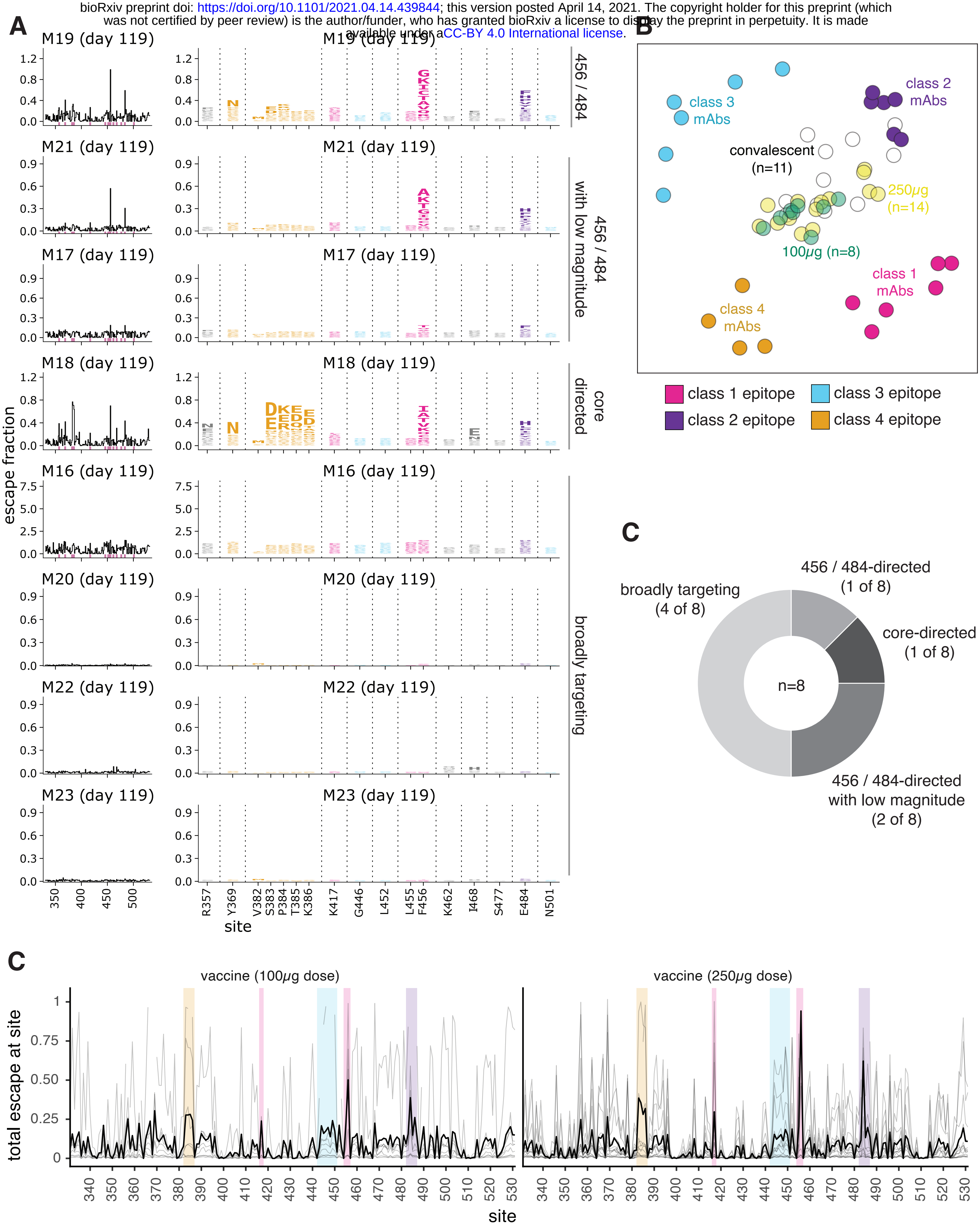

bioRxiv preprint doi: https://doi.org/10.1101/2021.04.14.439844; this version posted April 14, 2021. The copyright holder for this preprint (which was not certified by peer review) is the author/funder, who has granted bioRxiv a license to display the preprint in perpetuity. It is made available under aCC-BY 4.0 International license.

Fig. S6. Escape maps from individuals who received the 100 μg dose of mRNA-1273 119 days post-vaccination largely resemble those of individuals who received the 250 μg dose. (A) Logo plots representing the complete escape maps for the day 119 sera from 8 individuals who received the 100 μg dose of mRNA-1273. RBD sites are colored according to epitope, as in **Fig. 2A**. **(B)** Multidimensional scaling projection that illustrates relationships among escape maps of sera and monoclonal antibodies in two dimensions, similarly to the projection shown in **Fig. 4**. Similar mutations affect the binding of antibodies or sera located near one another in the plot. Here, the only serum samples shown are the day 119 samples for individuals who received the 100 or 250 μg vaccine dose. The 100 μg dose samples are shown in green and the 250 μg dose samples are shown in yellow (day 119 for all). The projection includes the escape maps of 22 monoclonal antibodies (escape maps first described in (*16*, *22*, *25*–*27*) of the 4 major structural classes to orient the plot. Antibodies are colored according to epitope, as in **Fig. 2A**. **(C)** Composite line plots showing the total binding escape at each site in the RBD for each sample in gray, and the mean site-total escape for each group (100 or 250 μg vaccine doses) as a thicker black line. 100 μg, n=8; 250 μg, n=14. The same key sites within each epitope are highlighted, as in **Fig. 4**.

bioRxiv preprint doi: https://doi.org/10.1101/2021.04.14.439844; this version posted April 14, 2021. The copyright holder for this preprint (which was not certified by peer review) is the author/funder, who has granted bioRxiv a license to display the preprint in perpetuity. It is made available under aCC-BY 4.0 International license.

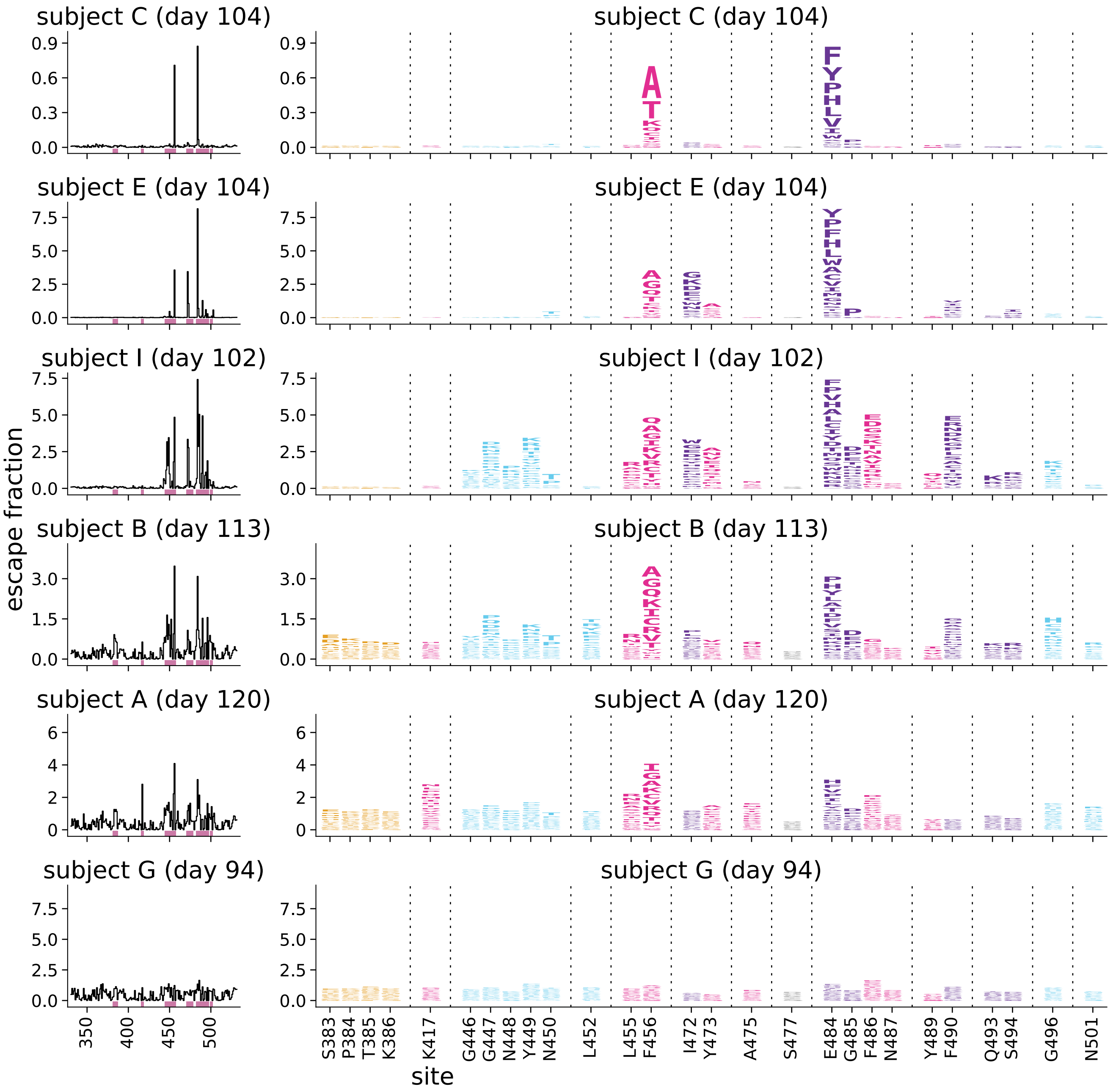

Fig. S7. Complete escape maps six representative convalescent plasmas from the day 100–150 cohort. RBD sites are colored according to epitope, as in **Fig. 2A** . The same sites are shown here as in **Fig. 2A** . These six plasmas are those used in neutralization assays shown in **Fig. 5, S 8, S 9**. Escape maps were first reported in (*15*).

bioRxiv preprint doi: https://doi.org/10.1101/2021.04.14.439844; this version posted April 14, 2021. The copyright holder for this preprint (which was not certified by peer review) is the author/funder, who has granted bioRxiv a license to display the preprint in perpetuity. It is made available under aCC-BY 4.0 International license.

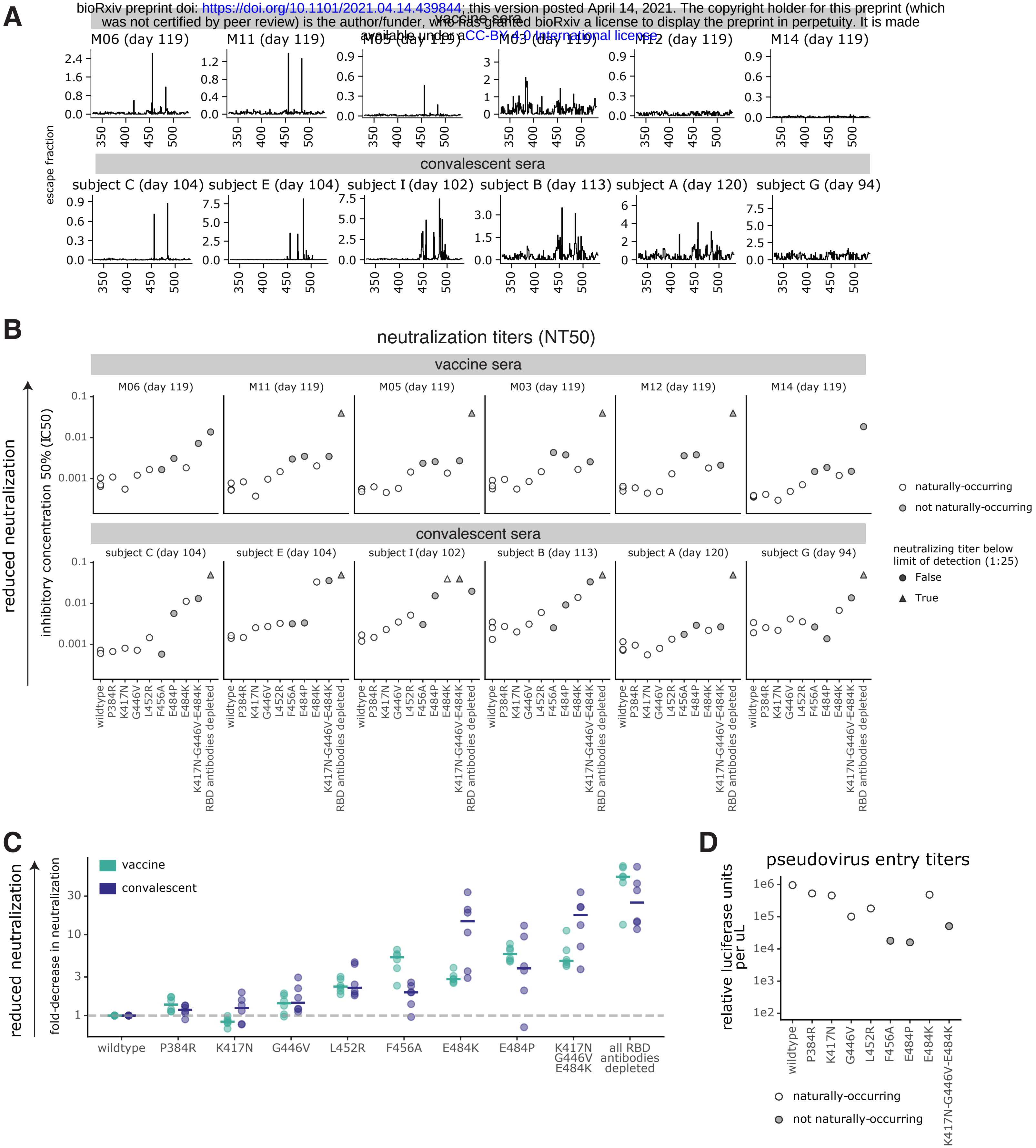

bioRxiv preprint doi: https://doi.org/10.1101/2021.04.14.439844; this version posted April 14, 2021. The copyright holder for this preprint (which was not certified by peer review) is the author/funder, who has granted bioRxiv a license to display the preprint in perpetuity. It is made available under aCC-BY 4.0 International license.

Fig. S8. Escape maps and effects of individual RBD mutations on neutralization for representative samples from vaccinated and convalescent individuals. (A) The site-wise antibody-binding escape for each of the vaccine and convalescent samples tested in neutralization assays in panels B and C and Fig. 5. **(B)** The effects of RBD mutations on neutralization of G614 spike-pseudotyped lentiviral particles with the indicated mutations, shown as the inhibitory concentration 50% (IC50). Naturally-occurring mutations are colored in white, and non-naturally-occurring mutations in gray. **(C)** The fold-change in IC50 compared to wild type spike, grouped by vaccine or convalescent sera (as in Fig. 5C, but shown here for all RBD mutants). Dashed line indicates no change in neutralization relative to wild type spike. Horizontal bars represent the group median fold-change IC50. In (B) and (C), each point represents the IC50 from one individual calculated from technical duplicates. The highest two points for E484K and K417N-G446V-E484K, and the highest 4 points for "all RBD antibodies depleted" are at the limit of detection. **(D)** Spike-pseudotyped lentiviral particle entry titers for RBD mutants tested in neutralization assays, calculated as the mean relative luciferase units per μL from 16 technical replicates. Mutations that are observed in at least one SARS-CoV-2 sequence in GISAID are colored in white, and non-naturally-occurring mutations in gray. All spike sequences contained G614, which fixed in circulating sequences in 2020 (*47*). All full neutralization curves are in **Fig. S9** and raw IC50 and NT50 values are at https://github.com/jbloomlab/SARS-CoV-2-RBD_MAP_Moderna/blob/main/experimental_data/results/mutant_neuts_results/fitparams.csv.

bioRxiv preprint doi: https://doi.org/10.1101/2021.04.14.439844; this version posted April 14, 2021. The copyright holder for this preprint (which was not certified by peer review) is the author/funder, who has granted bioRxiv a license to display the preprint in perpetuity. It is made available under aCC-BY 4.0 International license.

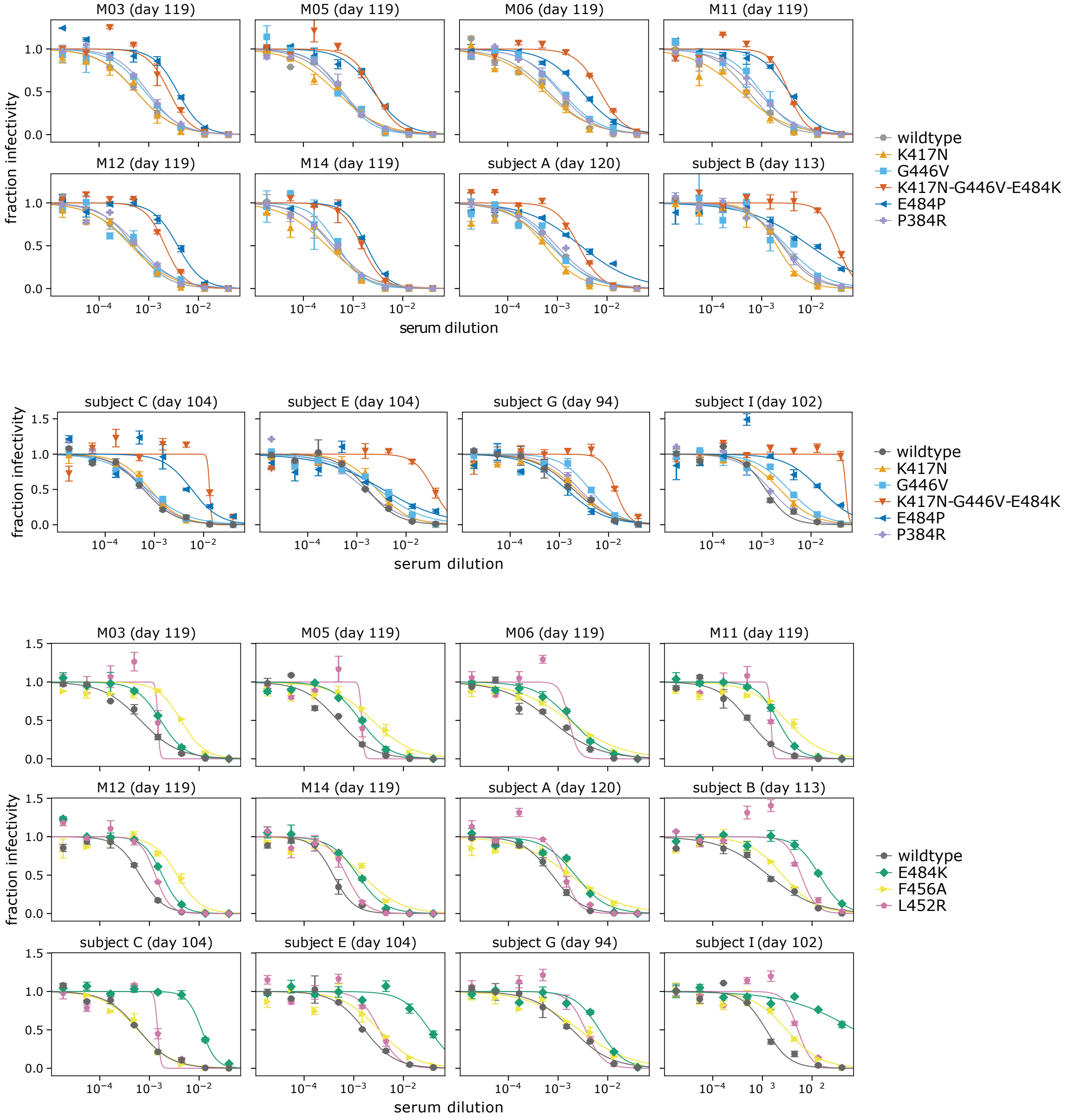

Fig. S9. Full neutralization curves for all assays testing how RBD mutations affected viral neutralization. The x-axis is the serum dilution, and the y-axis is the fraction of viral infectivity remaining at that dilution. The neutralization curves were fit and plotted using neutcurve (https://jbloomlab.github.io/neutcurve/ , version 0.5.2). IC50s were calculated by fitting 2-parameter Hill curves with the baselines fixed at one and zero. These IC50s were used to determine the fold-change values in **Fig. 5** and **S8**. In each plot, mutants are shown with the wildtype tested on the same date. Error bars represent the standard error of n=2 replicates. For readability, no more than 6 curves are shown per plot. In **Fig. 5D**, the wildtype curve from the first assay date is shown. Neutralization titers are available at https://github.com/jbloomlab/SARS-CoV-2-RBD_MAP_Moderna/blob/main/experimental_data/results/mutant_neuts_results/fitparams.csv

bioRxiv preprint doi: https://doi.org/10.1101/2021.04.14.439844; this version posted April 14, 2021. The copyright holder for this preprint (which was not certified by peer review) is the author/funder, who has granted bioRxiv a license to display the preprint in perpetuity. It is made available under aCC-BY 4.0 International license.

Supplementary Files:

Table S1. Serum neutralization titers pre- and post-depletion of RBD-binding antibodies. This file contains the serum neutralization titers from vaccinated individuals before (NT50_pre) and after (NT50_post) depletion of RBD-binding antibodies. The table is available online at https://github.com/jbloomlab/SARS-CoV-2-RBD_MAP_Moderna/blob/main/experimental_data/results/rbd_absorptions/TableS1.csv.

Table S2. Information on FACS sorting to select cells expressing RBD mutants with reduced binding by sera from vaccinated individuals. The file gives the number of antibody-escaped cells collected per selection for each replicate library and the percent of RBD+ cells in the antibody-escape gate for each selection, and the exact dilution used for each serum selection. The file is also available on GitHub at https://github.com/jbloomlab/SARS-CoV-2-RBD_MAP_Moderna/blob/main/data/TableS2_FACSinfo.csv.

Table S3. Measurements of effects of all amino-acid mutations to the RBD on serum binding.
The file gives the "escape fraction" for each mutation, as well as the total escape fraction at each site and the maximum escape fraction for any mutation at the site. This file includes escape fractions for sera from individuals vaccinated with mRNA-1273 as well as the previously reported escape fractions for convalescent plasma (*15*, *16*). The file is also available on GitHub at https://github.com/jbloomlab/SARS-CoV-2-RBD_MAP_Moderna/blob/main/results/supp_data/moderna_convalescent_all_raw_data.csv.
